# Mutating RBF Can Enhance Its Pro-Apoptotic Activity and Uncovers a New Role in Tissue Homeostasis

**DOI:** 10.1371/journal.pone.0102902

**Published:** 2014-08-04

**Authors:** Cécile Milet, Aurore Rincheval-Arnold, Angéline Moriéras, Amandine Clavier, Alexandrine Garrigue, Bernard Mignotte, Isabelle Guénal

**Affiliations:** Laboratoire de Génétique et Biologie Cellulaire - EA4589, Université de Versailles Saint-Quentin-en-Yvelines, Ecole Pratique des Hautes Etudes, Montigny-le-Bretonneux, France; IISER-TVM, India

## Abstract

The tumor suppressor retinoblastoma protein (pRb) is inactivated in a wide variety of cancers. While its role during cell cycle is well characterized, little is known about its properties on apoptosis regulation and apoptosis-induced cell responses. pRb shorter forms that can modulate pRB apoptotic properties, resulting from cleavages at caspase specific sites are observed in several cellular contexts. A bioinformatics analysis showed that a putative caspase cleavage site (TELD) is found in the Drosophila homologue of pRb (RBF) at a position similar to the site generating the p76Rb form in mammals. Thus, we generated a punctual mutant form of RBF in which the aspartate of the TELD site is replaced by an alanine. This mutant form, RBF^D253A^, conserved the JNK-dependent pro-apoptotic properties of RBF but gained the ability of inducing overgrowth phenotypes in adult wings. We show that this overgrowth is a consequence of an abnormal proliferation in wing imaginal discs, which depends on the JNK pathway activation but not on wingless (wg) ectopic expression. These results show for the first time that the TELD site of RBF could be important to control the function of RBF in tissue homeostasis *in vivo*.

## Introduction

The Retinoblastoma gene, *Rb*, was first identified as the tumor suppressor gene mutated in a rare childhood eye cancer, and its product (pRb) is often functionally inactivated in many human cancers by mutation or hyperphosphorylation [1], [2]. pRb is a member of the pocket protein family. These proteins possess specific A and B domains that form the pocket domain, required for their interactions with many transcription factors or co-factors in order to modulate the transcription of various genes (reviewed in [3], [4]). One of the major roles of pRb is to inhibit cell cycle progression by repressing the transcription of genes required for the G1-S transition, such as *cyclin E* or genes necessary for DNA synthesis, through binding and regulation of the E2F/DP transcription factors [5]. In addition, Rb has also been involved in chromosome dynamics during the M phase (rewieved in [6]).

Besides its roles on cell cycle, pRb regulates a variety of cellular processes, including angiogenesis, senescence, differentiation and apoptosis [7], [8]. In opposition to its well-established effects on cell cycle regulation, pRb role in apoptosis appears to be complex. Indeed, on the one hand pRb inactivation, partly by increasing free E2F1 DNA binding activity can induce cell cycle S phase entry and apoptosis, involving p53-dependent or –independent pathways [9], [10], which shows that pRb can be anti-apoptotic. On the other hand, several studies have shown that pRb can also be pro-apoptotic in several cellular contexts [11], [12], [13], [14], [15], [16], [17]. Although it has been shown that pRb localizes to mitochondria [18] where it induces apoptosis directly [19], little is known about the mechanisms that regulate pRb apoptotic functions. Many studies in mammals cells have shown that the pRb protein can be cleaved at several sites during apoptosis [20], [21], [22], [23], [24]. A cleavage at the C-terminus of pRb generates the p100^Rb^ form [25], and a more internal cleavage generates two forms: p48^Rb^ and p68^Rb^ [26]. These cleavages are realized by specific caspases at consensus cleavage sites ending by an aspartate [25], [26]. In addition, we previously described another cleavage of pRb by caspase 9 at a LExD site, which generates the p76^Rb^ form [13]. These cleavages are certainly part of a poorly understood regulation process of pRb functions. Indeed, pRb cleavage can often be observed when apoptosis is induced by different ways, and impairing pRb C-terminus cleavage reduces apoptosis [23], [27]. We have shown that p76^Rb^ is pro-apoptotic in several human cell lines [28] but possesses anti-apoptotic properties in rat embryonic fibroblasts [13]. This discrepancy could be related to a differential regulation of apoptotic genes by pRb in human versus rodent cells [29]. Altogether, these results show that cleavage of pRb can exert specific activities on the control of apoptosis.

*Drosophila* is a powerful model for genetic studies, which can be used to better understand apoptosis regulation by pocket proteins, and modes of regulation of these proteins *in vivo*. Components of the E2F/pRb pathway are highly conserved and simpler in *Drosophila* than in mammals. In *Drosophila*, only one DP (dDP), two E2F proteins (dE2F1, dE2F2) and two pRb family proteins (RBF, RBF2) have been described [30], [31]. As in mammals, RBF binds to and inhibits the transcription factor dE2F1 [32], thus impairing its ability to induce transcription of genes whose products are necessary for cell cycle progression, like *cyclin E* [33]. In *Drosophila*, loss of function clones for *RBF* display an increased sensitivity to irradiation-induced apoptosis [34], [35], [36], and an increased level of apoptosis is observed in *RBF^−/−^* embryos [37]. According to these studies, RBF has an anti-apoptotic role in *Drosophila*. Despite this prevalent view, we have recently shown that RBF can also exert a pro-apoptotic effect in proliferating cells, in a caspase-dependent manner [38], which is more in acquaintance with its tumor-suppressor role. Thus, the complexity of pRb effects on apoptosis is conserved in *Drosophila*.

In this paper, we identified a TELD site in RBF sequence, which is most probably equivalent to the LExD site of mammalian pRb. In order to determine if RBF TELD site can modulate RBF properties on apoptosis, we generated a mutant form of RBF, RBF^D253A^, in which the aspartate of the cleavage site, which is necessary for caspase recognition, is switched into an alanine. We observed that RBF^D253A^ expression remains pro-apoptotic in proliferating cells of the wing imaginal disc, the adult wing primordium, and that this process depends on the activation of JNK pathway. Interestingly, *RBF^D253A^* expression also induces ectopic proliferation and overgrowth in the wing tissue, which also depend on the JNK pathway but not on *wg* ectopic expression. This overgrowth was never observed when *RBF* was expressed. Therefore, mutating the TELD caspase cleavage site modulates the properties of RBF and affects tissue homeostasis. This result indicates that RBF cleavage by caspases *in vivo* could be important to control its effects on cell fate during development.

## Materials and Methods

### Fly stocks and breeding conditions

Flies were raised on standard medium. The *UAS-RBF* and *vg-Gal4* strains were generous gifts from J. Silber. The *en-Gal4*, *ptc-Gal4* and *UAS-EGFP* strains were generous gifts from L. Théodore. The *C96-Gal4 strain* was a generous gift from F. Agnes. The *UAS-bsk-RNAi* strain was a generous gift from S. Netter. The *UAS-mtGFP*, *UAS-p35* and the *hep^r75^* strains come from the Bloomington stock center, and the *UAS-wg-RNAi* strain comes from the Vienna Drosophila Resource Center. A Canton S *w^1118^* line was used as the reference strain. All crosses using the Canton S *w^1118^*, *UAS-RBF* and *UAS-RBF^D253A^(B18)* strains were performed at 25°C and all crosses using the *UAS-RBF^D253A^(B2.3)* were performed at 21°C to induce production of similar protein levels. In these conditions both lines exhibit similar phenotypes.

### Generation of transgenic flies

The *rbf* full-length cDNA was provided by N. Dyson. We generated the non-cleavable form of RBF by changing the aspartate 253 to an alanine. Mutagenesis was conducted with the Quikchange Site-Directed Mutagenesis kit (Stratagen#200518) by using sens RBFmut903 5′CTGGACGGAGCTG**GCA**TTTCGTCACAATCCG3′ and antisens RBFmut903 5′CGGATTGTGACGAAA**TGC**CAGCTCCGTCCAG3′ primers. The Not1/Kpn1 insert was then subcloned into the pUAST vector to produce the pUAST-RBF^D253A^ vector and sequenced to verify its integrity. The pUAST-RBF^D253A^ construct was injected into Canton S *w^1118^* fly embryos following standard procedures to obtain transgenic *Drosophila* strains. Independent transgenic lines were characterized and used for further experiments.

### TUNEL staining of imaginal discs

*C96-Gal4, vg-Gal4* and *ptc-Gal4* females were crossed with *w^1118^*, *UAS-RBF*, *UAS-RBF^D253A^* or *UAS-bsk-RNAi* males for apoptosis detection. Wing imaginal discs of the progeny were dissected in PBS pH 7.6, fixed in PBS/formaldehyde 3.7%, washed three times for 20 min in PBT (1X PBS, 0.5% Triton). Discs were then dissected, TUNEL staining was performed following manufacturer's instructions (ApopTag Red *in situ* apoptosis detection kit, Chemicon), and discs were mounted in CitifluorTM (Biovalley) and observed with a conventional Leica DMRHC research microscope using the N2.1 filter. For quantification experiments, discs were observed with a Leica SPE upright confocal microscope. White patches in the wing pouch were counted for at least 30 wing imaginal discs per genotype. Student's tests were performed and results were considered to be significant when α<5%.

### Histochemistry

The following antibodies were used: anti-RBF (rabbit polyclonal anti-RBF, 1∶500, Custom antibody), anti-Wg (mouse monoclonal antibody, DSHB, clone number 4D4, 1∶100), anti-◯P-JNK (rabbit polyclonal antibody, 1∶500, Promega number V7931). Third instar larvae were dissected in PBS pH 7.6, fixed in PBS/3.7% formaldehyde, washed three times for 20 min each in PBT (1X PBS, 0.3% Triton) and incubated with the primary antibody overnight at 4°C in PBT/FCS (1X PBS, 0.3% Triton, 10% FCS). Incubation with the secondary antibody was carried in PBT/FCS for 2 hours at room temperature. Larvae were then washed thrice in PBT and placed in PBS/glycerol (1∶1) overnight at 4°C. Finally, discs were mounted in Citifluor™ (Biovalley) and observed with a conventional Leica DMRHC research microscope, using the L5 filter to detect green fluorescence, the N2.1 filter for the red fluorescence.

### BrdU labeling of wing discs

*ptc-GAL4* or *en-GAL4* females were crossed with *UAS-RBF* and *UAS-RBF^D253A^* males, and with *w^1118^* males for the control. Larvae were fed for 2 h on medium supplemented with 1 mg/ml BrdU. They were then dissected in 1X PBS pH 7.6, and fixed in PBT/formaldehyde (PBS 1X/5% formaldehyde/0.3% Triton X-100) for 20 min at room temperature, washed three times for 31min in PBT, denatured in 2.2 N HCl/0.1% Triton X-100 by two 15 minute-long incubations, neutralized with 100 mM sodium tetraborate (Borax) by two 5 minute-long incubations. For immunohistochemistry, larvae were blocked by incubation in PBT/10% fetal calf serum (FCS) for 45 min, incubated with mouse anti-BrdU monoclonal antibody (1: 200, DSHB) overnight at 4°C and washed thrice in PBT/FCS. Discs were then incubated with anti-mouse IgG-FITC (1: 200, Jackson Immuno Research) and washed thrice in PBT. Discs were mounted in Citifluor™ and observed with a Leica SP2 upright confocal microscope.

## Results

### Generation of RBF^D253A^, a mutant form of RBF affecting a consensus conserved caspase cleavage site

We have used the CASVM web server [39] to scan the full length RBF for potential caspase cleavage sites predicted by the support vector machines (SVM) algorithm [40]. This was done with the P14P10′ window (tetrapeptide cleavage sites with ten additional upstream and downstream flanking sequences) which has the highest accuracy. Using this window, only one caspase cleavage site was found in RBF (Figure S1A in File S1). This unique conserved cleavage site, TELD, fulfills the criteria of substrate specificity reported for Dronc, the *Drosophila* homologue of Caspase 9 [41], [42]. Furthermore, the TELD sequence is located in position 253 of RBF in the region containing the LExD site that leads to the generation of the p76Rb form in mammals [13] and a consensus caspase cleavage site, LEND, can also been found in the *C. elegans* homolog at the same position (Figure S1B in File S1). The conservation of a caspase cleavage site through evolution suggests a physiological role for RBF cleavage in this region including in *Drosophila*.

Using western blot analysis we were unable to consistently observe a band at the expected apparent molecular weight of the cleaved form. We explain this difficulty by the incapacity of the only available antibody to reveal cleaved forms. To by-pass this problem, vectors allowing the expression of C-terminal HA-tagged forms of RBF and RBF^p76^, the C-terminal part of *RBF* expected to result from a cleavage at the putative caspase site were transfected into S2 cells. Western blot experiments reveal the presence of cleaved forms of RBF (Figure S1C in File S1). One of these has the expected size of RBF^p76^. This result shows that RBF can be cleaved in the TELD region.

As caspases recognize specific motifs ending by an aspartate, we generated a mutant form of RBF, RBF^D253A^, in which aspartate 253 is switched into an alanine. This mutation should impair a cleavage of the RBF protein at the TELD site by caspases. This kind of approach was successfully conducted in mammals to generate a cleavage-resistant form of pRb at a C-terminus consensus cleavage site by caspases [25]. Indeed, the expression of the HA-tagged form of *RBF^D253A^* in S2 cells does not seem to generate the cleaved form (Figure S1C in File S1). In order to determine if the RBF consensus caspase cleavage site could have a physiological relevance in regulating RBF functions *in vivo*, we generated several independent transgenic fly strains carrying the *RBF^D253A^* mutant form under control of the *UAS* transcription regulating sequence.

### Expression of RBF or RBF^D253A^ induces different phenotypes

To determine if the mutation of the TELD site modifies RBF activity, we tested if expression of *RBF* and *RBF^D253A^* could result in different adult phenotypes. Since the random insertion locus of transgenes in *Drosophila* transgenic strains can induce different expression rates of a same transgene in independent transgenic strains, we have studied two independent *RBF^D253A^* transgenic strains. The phenotypes were similar for both transgenic lines (data not shown). To compare *RBF* and *RBF^D253A^* effects, we first verified by RT-qPCR and western blot that *RBF* mRNA level and full-length RBF protein rates were similar in RBF and RBF^D253A^ transgenic strains (Figure S2 in File S1).

**Figure 1:**
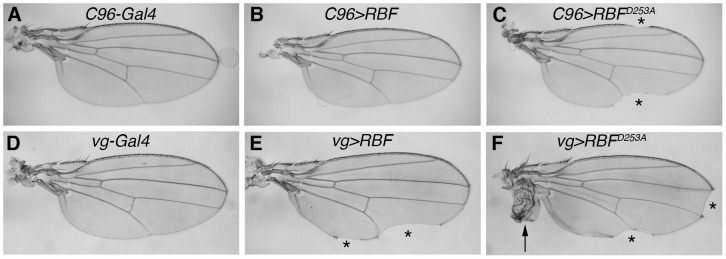
RBF and RBF^D253A^ induce different phenotypes in adult wings. Transgenes were expressed in non-proliferating cells of the ZNC during wing development (A–C) or proliferating cells along the dorso-ventral boundary (D–F). (A) *C96-Gal4/+* control wings show a continuous wing margin. (B) *C96-Gal4/UAS-RBF* adult wings are similar to control wings. (C) In *UAS-RBF^D253A^*/+; *C96-Gal4/+* flies, *RBF^D253A^* induces notches at the wing margin (asterisks). (D) *vg-Gal4/+* control wings display a continuous wing margin. (E) In *vg-Gal4/+*; *UAS-RBF/+* flies, *RBF* expression induces notches at the wing margin (asterisks). (F) In *vg-Gal4/UAS-RBF^D253A^* flies, *RBF^D253A^* expression provokes not only notches (asterisks) but also the apparition of hyperplastic tissue in the wing (arrow).

Since our previous results have shown that RBF expression is pro-apoptotic in cycling cells whereas it is not in post-mitotic cells [38], we used various wing specific drivers allowing an expression in cycling or non-cycling cells of the wing imaginal disc. In agreement with our previous results, we observed that RBF expression in non-cycling cells of the dorso-ventral boundary (ZNC) did not affect wing development, as adult *C96>RBF* wings showed wild type phenotype similar to *C96-Gal4/+* control wings (Fig. 1, A, B). On the contrary, *C96>RBF^D253A^* wings presented notches at their margin (Fig. 1 C, asterisks) that resulted from tissue loss. Thus, the mutant and wild-type forms of RBF display different properties in non-cycling cells, which indicates that mutating the TELD site modifies RBF properties in these cells.

We have previously shown that *RBF* expression in cycling cells, under *vg-Gal4* driver, generates notches in the wing margin, which result from RBF induced-apoptosis during third larval instar in the *vg-Gal4* expression domain of the wing imaginal disc. To test the effects of *RBF^D253A^* expression in proliferating wing cells, we also used the *vg-Gal4* driver. Expression of *RBF* under control of the *vg-Gal4* driver led to notches at the wing margin (Fig. 1E, asterisks) and *RBF^D253A^* conserved this property (Fig. 1 F). Strikingly, using several independent transgenic strains, we also observed a phenotype of ectopic tissue next to the hinge of the wing, in up to 30% of the *vg>RBF^D253A^* flies (Fig. 1F, arrow). This phenotype is probably the consequence of abnormal cell proliferation and is specific of the RBF^D253A^ form as it was never observed when wild type *RBF* was expressed at equivalent levels.

Finally, we tested the effects of ubiquitous expression of *RBF* and *RBF^D253A^* under control of the *da-Gal4* driver. Such expression of *RBF* at 25°C induced notches on the wing margin (data not shown). Ubiquitous expression of *RBF^D253A^* was lethal when flies were raised at 25°C as well as at lower temperature (21°C) to reduce RBF^D253A^ quantity. In contrast, *RBF* ubiquitous expression never revealed lethal, even at higher levels when flies were raised at 29°C.

Altogether, these results show that RBF^D253A^ properties differ from RBF properties, both in cycling and non-cycling cells during development. Therefore, a cleavage of RBF at the TELD site may be important to regulate its functions in different cellular contexts.

### RBF^D253A^ induces more apoptosis than RBF in the wing imaginal disc

**Figure 2:**
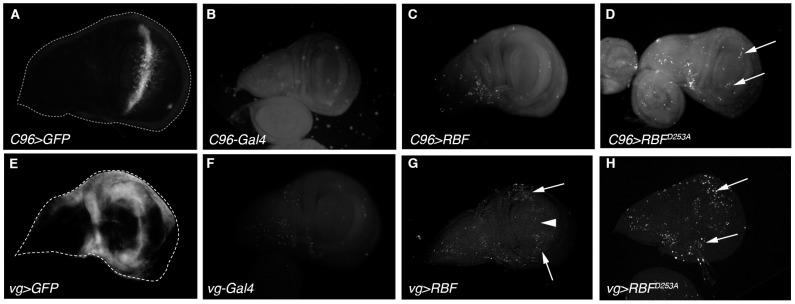
RBF^D253A^ is pro-apoptotic in the ZNC and induces more apoptosis than RBF in third instar larvae wing imaginal discs. (A, E) *C96-Gal4* and *vg-Gal4* expression patterns are visualized by *UAS-mtGFP* expression in third instar larvae wing imaginal discs. (B-D, F-H) Apoptotic cells are labeled by TUNEL in wing imaginal discs; specific staining of apoptotic cells corresponds to bright white patches. (B, F) *C96-Gal4/+* and *vg-Gal4/+* control discs have few apoptotic cells. (C) *C96-Gal4/UAS-RBF* wing discs are similar to control. (D) Some apoptotic cells are observed within the *C96-Gal4* expression domain in *UAS-RBF^D253A^/X*; *C96-Gal4/+* discs (white arrow). (G, H) Apoptotic cells are observed within the *vg-Gal4* expression domain in *vg-Gal4/+*; *UAS-RBF/+* and *UAS-RBF^D253A^/X*; *vg-Gal4/+* wing discs (white arrows). (G) The white arrowhead indicates a zone at the center of the pouch where cells are not TUNEL-labeled in *vg-Gal4/+*; *UAS-RBF/+* wing discs. All discs are shown with posterior to the top.

We have previously shown that *RBF* expression in proliferative cells induces apoptosis, including in the wing tissue. To check if RBF^D253A^-induced notch phenotype was also correlated with apoptosis induction, we performed TUNEL staining on larvae wing imaginal discs expressing *UAS-RBF* or *UAS-RBF^D253A^* under the control of *C96-Gal4* or *vg-Gal4* drivers (Fig. 2). The expression domains of these drivers were visualized in control wing discs by inducing *UAS-mtGFP* (Fig. 2A,E). In *C96-Gal4* and *vg-Gal4* control discs (Fig. 2B and F), developmental apoptosis was rare (few white bright dots). In *C96>RBF* wing discs (Fig 2 C), TUNEL staining was similar to control discs, which shows that RBF does not induce apoptosis in cells of the ZNC, in acquaintance with the absence of notches in *C96>RBF* adult wings (Fig 1 B). On the contrary, in *C96>RBF^D253A^* wing discs, some cells located at the center of the pouch in the zone corresponding to the ZNC were TUNEL labeled (Fig 2 D, arrows). Similar results were observed using Acridine Orange (AO) staining (Figure S3 in File S1) indicating that RBF^D253A^ expression induces apoptosis in this region, leading to the appearance of notches in adult wings (Fig 1 C). In *vg*>*RBF* and *vg>RBF^D253A^* wing discs, apoptotic cells were observed within the driver expression domains (Fig. 2 G, H white arrows). Interestingly, RBF did not induce apoptosis at the center of the pouch in *vg>RBF* discs (Fig 2 G, arrow head) whereas RBF^D253A^ is pro-apoptotic in this area that includes the ZNC. These observations are coherent with the fact that, contrarily to RBF, RBF^D253A^ is pro-apoptotic in non-proliferating cells as observed with the *C96-Gal4* driver.

**Figure 3:**
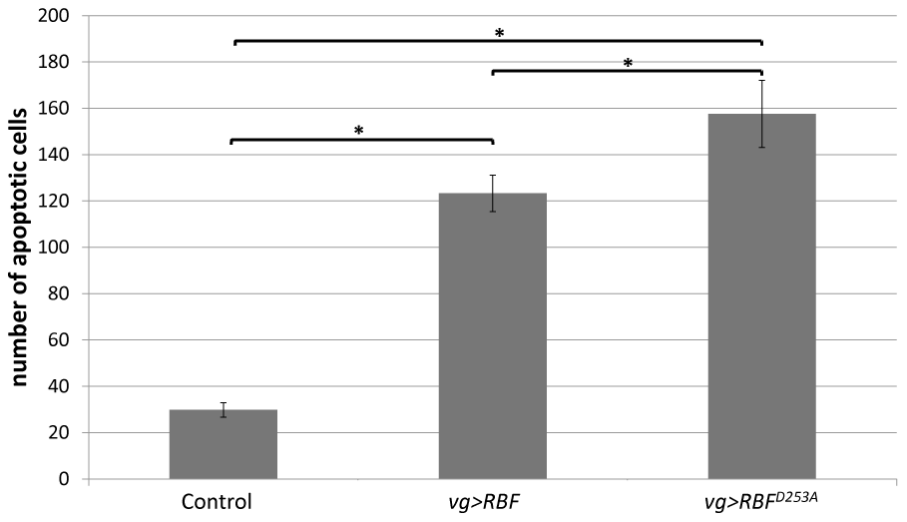
RBF^D253A^ is more pro-apoptotic than RBF in third instar larvae wing imaginal discs. Apoptotic cells were visualized by TUNEL staining of wing imaginal discs of *vg-Gal4/+*; *UAS-RBF/+* or *vg-Gal4/UAS-RBF^D253A^* genotypes. TUNEL positive cells in the wing pouch were quantified. Asterisks indicate a statistically significant difference between two genotypes (Student's test, p<0,05).

We also tested the effects of *RBF* and *RBF^D253A^* expression in cycling cells in a different and more restricted expression domain. Using *ptc-Gal4* we drove *RBF* and *RBF^D253A^* expression at the antero-posterior boundary of the wing imaginal disc. Similar experiments on *ptc>RBF* and *ptc>RBF^D253A^* wing discs also showed that *RBF* and *RBF^D253A^* expression was associated with apoptosis (Figure S4 in File S1), confirming that both forms are pro-apoptotic in proliferating cells. In these experiments using *vg-Gal4* and *ptc-Gal4* to drive the expression of both RBF forms, it seemed that more cells were apoptotic in *RBF^D253A^* -expressing discs. To test if RBF^D253A^ induced significantly more apoptosis than RBF, we have quantified TUNEL staining of *vg>RBF* or *vg>RBF^D253A^* wing imaginal discs. Staining patches in the wing pouch were counted for at least 30 imaginal discs per genotype (Fig 3). We observed that RBF^D253A^ induced significantly more apoptosis than RBF (α<5%).

### RBF^D253A^ induces increased proliferation of neighboring cells

**Figure 4:**
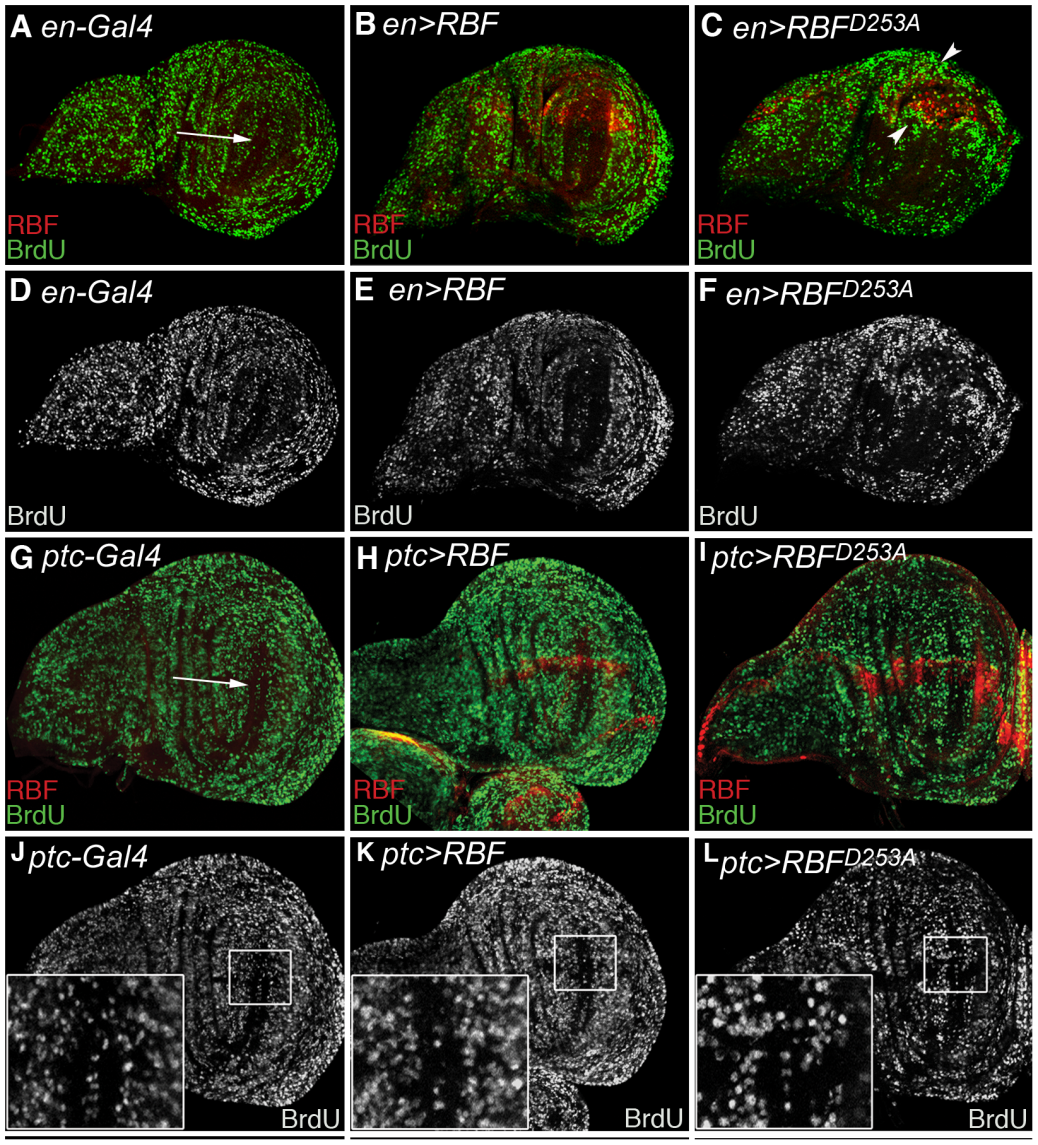
*RBF^D253A^* expression induces proliferation of neighboring cells. (A–F) From left to right, phenotypes of the larvae are: *en-Gal4/+*, *en-Gal4/+*; *UAS-RBF/+* and *UAS-RBF^D253A^X; en-Gal4/+*. (A–C) S phase staining by BrdU (green), and RBF immuno-staining (red). (D–F) BrdU staining (white) of the discs shown in (A–C). (B, E) In RBF expressing discs, as in the control disc shown in (A,D), BrdU staining is homogeneous in the whole disc, except in the ZNC (Zone of Non-proliferating Cells) (white arrow). (C–F) In RBF^D253A^ expressing discs, cells surrounding the strong RBF staining exhibit an enhanced BrdU staining, indicating that these cells have an increased proliferation rate. (G–L) From left to right, genotypes of the larvae are: *ptc-Gal4/+*, *ptc-Gal4/UAS-RBF*, and *UAS-RBF^D253A^* X; *ptc-Gal4/+*. (G–I) S phase staining by BrdU (green), and RBF staining (red). (J–L) BrdU staining (white) of the discs shown in (G–I) with enlarged view of boxed area. (G, J) BrdU staining is homogeneous in the whole disc, except in the ZNC (white arrow). (I–L) In *ptc>RBF^D253A^* discs, cells within the ZNC that are adjacent to RBF^D253A^ expressing cells are labeled with BrdU, indicating an abnormal proliferation of these cells. All discs are shown with posterior to the top.

In order to check if the overgrowth observed in wings of RBF^D253A^ expressing flies was due to an enhanced proliferation, we performed a BrdU incorporation assay to label cells in the S phase of the cell cycle in wing imaginal discs. As this tissue is highly proliferative, BrdU incorporation occurred throughout the whole disc. Only a small population of cells is arrested in the cell cycle and forms the ZNC, which is situated at the center of the wing pouch (Fig. 4A,G, white arrows). We used the *en-Gal4* driver to express *RBF* forms in the posterior part of the discs, allowing the use of the anterior compartment as an internal control. Over-expression of *RBF* and *RBF^D253A^* was visualized by RBF staining (red) (Fig. 4B, C). The BrdU incorporation profile was similar in both *RBF*-expressing and control discs (Fig. 4A,B and D,E). On the contrary, a more intense BrdU staining was observed in the most posterior part of discs expressing *RBF^D253A^*, but also in cells located near the antero-posterior border, a region that did not express *RBF^D253A^* in these discs (Fig. 4C, see arrowheads). Therefore, *RBF^D253A^* expression specifically induces abnormal proliferation of cells in a non-autonomous way.

As *en-Gal4* is expressed throughout the whole posterior part of the discs, we used the *ptc-Gal4* driver to reduce possible alterations of the disc morphology when *RBF^D253A^* is expressed. In *ptc>RBF^D253A^* discs, *RBF^D253A^* is expressed along the antero-posterior boundary that crosses the ZNC at its center. Non-cycling cells of the ZNC do not incorporate BrdU, thus abnormal BrdU staining in these cells is easy to detect. In *ptc>RBF* wing discs, BrdU staining was similar to control discs (Fig. 4G,H). None of the cells of the ZNC, whether they expressed *RBF* or not, displayed abnormal staining (Fig. 4J,K boxed areas showing the intersection of the ZNC with the antero-posterior boundary). On the contrary, in *ptc>RBF^D253A^* discs, we observed BrdU-labeled cells within the ZNC in the posterior side. This abnormal proliferation was adjacent to RBF^D253A^-expressing cells (Fig. 4 I,L boxed area). We observed that these cells localized in the posterior compartment along the antero-posterior boundary expressed ectopically the dorso-ventral boundary marker *wg*, and thus normally belong to the ZNC (Figure S5 in File S1). Therefore, these observations suggest that cells of the ZNC that do not express RBF^D253A^ are induced to proliferate when this mutant form is expressed along the antero-posterior border of the disc. These results confirm that RBF^D253A^ expression is able to induce abnormal proliferation of neighboring cells, even in a domain in which cells are normally arrested in the G1 phase of the cell cycle.

It is well established that cells undergoing apoptosis in a growing tissue promote proliferation of surrounding healthy cells [43], maintaining tissue homeostasis by a process named apoptosis-induced proliferation [44], [45]. We have observed that both RBF and RBF^D253A^ could induce apoptosis in proliferating cells and that *RBF^D253A^* induced more apoptosis than *RBF* when expressed at a similar rate. As RBF^D253A^ enhanced the proliferation rate of cells adjacent to its expression domains and subsequent wing tissue overgrowth, we wondered if the apparition of ectopic tissue in the wing was a specific effect of *RBF^D253A^* expression, and not only a consequence of an increased apoptosis induced by this mutant form. For this reason, we tested if enhancing RBF-induced apoptosis could result in tissue overgrowth. As the UAS/Gal4 system efficiency depends on temperature, we increased the breeding temperature of *vg>RBF* flies from 25°C to 29°C to enhance *RBF* expression. We also elevated the dose of *UAS-RBF* by expressing two independent *p[UAS-RBF]* transgene insertions in the same flies. Under these conditions, the strength of the notch phenotype and the level of AO staining in wing discs were increased to the same level as what was observed with the RBF^D253A^ construct, but we never observed ectopic tissue in the wings (data not shown). Thus, the effect of RBF^D253A^ on proliferation is specific of this mutant form, which reinforces the view that RBF cleavage could be necessary to control its activities *in vivo*. It suggests that RBF^D253A^ amplifies an apoptosis-induced proliferation mechanism, leading to an excessive proliferation in response to apoptosis.

### Activity of the JNK pathway is necessary for RBF- and RBF^D253A^-induced apoptosis and for RBF^D253A^-induced overgrowth

**Figure 5:**
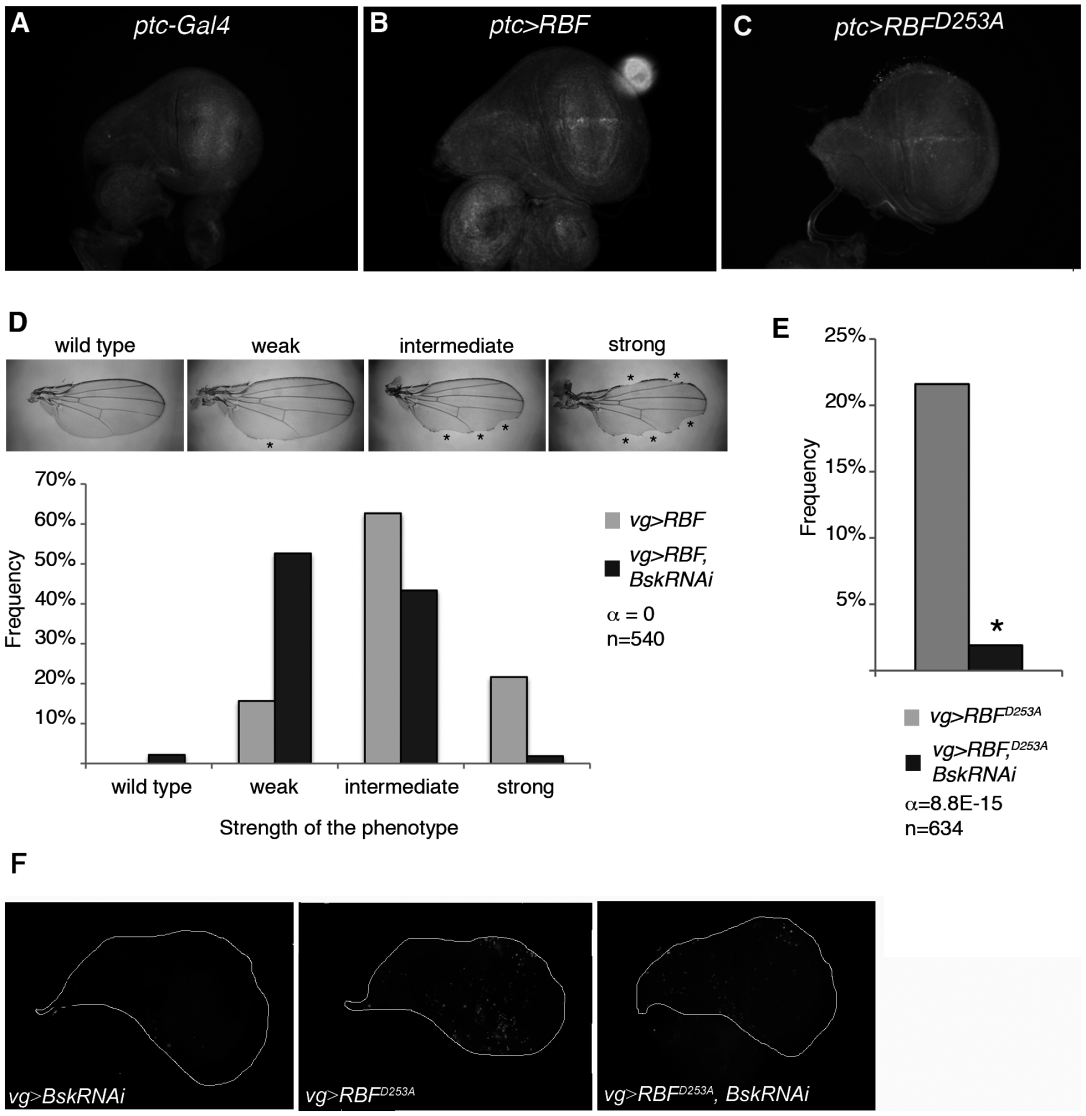
RBF- and RBF^D253A^-induced apoptosis as well as RBF^D253A^-induced overgrowth depend on the JNK pathway activity. (A–C) Anti-◯P-JNK staining in young third instar larvae wing imaginal discs. (A) No staining is observed in *ptc-Gal4/+* control. (B, C) *UAS-RBF/ptc-Gal4* and *UAS-RBF^D253A^/X; ptc-Gal4/+* discs present anti-◯P-JNK staining at the dorso-ventral boundary. All discs are shown with posterior to the top. (D) Notch phenotypes in adult wings of *vg-Gal4/+*; *UAS-RBF* and *vg-Gal4/+*; *UAS-RBF/UAS-bsk-RNAi* flies are grouped into four categories (wild type, weak, intermediate and strong) according to the number and size of notches observed on the wing margin (asterisks). *Bsk-RNAi* partially suppresses *RBF*-induced notch phenotypes (Wilcoxon test, α<10^−15^, n = 540). (E) The frequency of RBF^D253A^-induced ectopic tissue growth is strongly decreased in *UAS-bsk-RNAi* co-expressing flies (Chi^2^ test, α = 8.8 10^−15^). (F) TUNEL-labeling of apoptotic cells in wing imaginal discs; specific staining of apoptotic cells corresponds to bright patches in wing discs *of vg-Gal4/+*; *UAS-bsk-RNAi/+* (left panel), *vg-Gal4/+*; *UAS-RBF^D253A^/+* (center panel), *vg-Gal4/+*; *UAS-RBF^D253A^/UAS-bsk-RNAi* (right panel) *larvae*. RNAi-mediated knockdown of *bsk* strongly decreased RBF^D253A^-induced apoptosis. All discs are shown with posterior to the top.

The JNK pathway is implicated in both apoptotic and proliferation processes (reviewed in [46]). Among these processes, compensatory proliferation allows injured tissues to recover their original size by inducing ectopic proliferation of surviving cells. Moreover, in models involving cells induced to die by apoptosis but kept alive by the caspase inhibitor p35, the so-called “undead cells” emit persistent mitogen signaling that promotes overgrowth under control of the JNK pathway [43]. We thus tested if the JNK pathway was required for RBF- and RBF^D253A^-induced apoptosis and for RBF^D253A^-induced ectopic proliferation. The active form of the *Drosophila* JNK Bsk was stained with an anti-◯P-JNK antibody in *ptc>RBF* and *ptc>RBF^D253A^* wing discs. In *ptc-Gal4/+* control discs, we did not detect any specific staining in the *ptc* domain, which indicates that the JNK pathway is not activated in this domain during normal development (Fig. 5 A). On the contrary, in *ptc>RBF* and *ptc>RBF^D253A^* discs, we observed JNK activation in the *ptc-Gal4* expression domain, *i.e*. at the antero-posterior border of the discs (Fig. 5 B, C). This activation was strong in young third instar larvae, and decreased when larvae got older (data not shown).

To test if the JNK pathway was required for RBF-dependent apoptosis, we used a *p[UAS-bsk-RNAi]* transgene to disrupt the JNK pathway. Under *vg-Gal4*, the number and size of notches present at the wing margin is correlated with the amount of apoptosis [38]. We classified the wing phenotypes into four categories (wild type, weak, intermediate and strong) according to the number and size of notches (Fig. 5D, asterisks). In a control experiment, we verified that wings were wild type in *vg>bsk-RNAi* flies (data not shown). We assayed for the strength of the notch phenotype in wings of *vg>RBF* flies in presence or absence of *UAS-bsk-RNAi* (Fig. 5D). When *bsk-RNAi* was co-expressed with *RBF*, distribution of the phenotypes significantly shifted toward weaker phenotypes when compared to the expression of *RBF* alone (Wilcoxon test, α<10^−15^, n = 540) (Fig. 5D). We also disrupted the JNK pathway by using the *hep^r75^* mutant and observed that distribution of the RBF-induced notch phenotype was weaker in the *hep^r75^* mutant background (data not shown). These results clearly show that the JNK pathway is involved in RBF-induced apoptosis.

We also tested if the JNK pathway activation was required for RBF^D253A^-induced apoptosis and ectopic tissue. We counted the number of *vg>RBF^D253A^* flies presenting ectopic tissue in the wing in the presence or absence of *UAS-bsk-RNAi* and observed a strong decrease of their frequency: from 22.1% of *vg>RBF^D253A^* flies to only 1.9% of *vg>RBF^D253A^, bsk-RNAi* flies displaying overgrowth (Chi^2^ test, α = 8.8E-15) (Fig. 5E). We obtained similar results with *vg>RBF^D253A^* flies in a *hep^r75^* heterozygous background (data not shown). In parallel, we performed TUNEL staining in *vg>RBF^D253A^* wing imaginal discs in the presence or absence of *UAS-bsk-RNAi* (Fig. 5F), and detected less apoptotic cells when *bsk-RNAi* was co-expressed. Thus, similarly to RBF, RBF^D253A^-induced apoptosis depends on the activity of the JNK pathway.

In conclusion, we demonstrated that RBF and RBF^D253A^ activate the JNK pathway, that this pathway mediates both RBF and RBF^D253A^ -induced apoptosis, and is responsible for RBF^D253A^-induced overgrowth.

### RBF^D253A^-induced overgrowth does not depend on wg ectopic expression

As previously indicated, the JNK pathway is essential to both RBF^D253A^-induced overgrowth, and over-proliferation induced by “undead cells”. Its over-activation in “undead cells” leads to ectopic synthesis and secretion of the mitogenic proteins Wg and Dpp in a long lasting manner, leading to an over-proliferation of neighboring cells and overgrowth phenotypes [43]. Since RBF^D253A^-induced overgrowth depends on JNK pathway activation, we wondered if this process was provoked by a similar mechanism. We thus co-expressed *RBF* and *p35* in wing discs to generate undead cells depending on RBF-induced apoptosis, and compare the *wg* expression pattern in these discs to *wg* expression in RBF^D253A^ expressing discs. In *vg>RBF, p35* flies raised at 25°C, only one wing observed displayed overgrowth out of 123 flies counted. Therefore, even in the presence of p35, RBF does not seem to induce an overgrowth phenotype that would result from the presence of undead cells. However, at 29°C, more wings co-expressing RBF and p35 presented overgrowth phenotype (10 wings out of 35), but in such extreme conditions only few flies hatched. We tested if the Wg pattern was altered in these conditions as it has been observed in the presence of “undead cells”. We used the *en-Gal4* driver and compared the Wg staining in the control anterior compartment and in the posterior compartment that expresses RBF. *en>RBF* wing discs displayed a wild type Wg pattern similar to what was observed in *en-Gal/+* control discs (data not shown). In *en>RBF, p35* wing discs, the Wg pattern in the posterior compartment is altered when compared to control *en>p35* disc (Figure S6 panel D,E in File S1). Ectopic patches were observed outside the normal *wg* expression domain (Figure S6 panel E, white arrows in File S1) as was reported in studies of undead cells-induced overgrowth [47]. In *en>RBF^D253A^* discs, the *wg* expression pattern is also altered, but is different from what is observed in *en>RBF*, p35 discs. We did not observe any ectopic Wg patch in *en>RBF^D253A^* wing discs, but an enlargement of the Wg pattern in the posterior part of the disc (Figure S6 panel F in File S1). Thus, the *wg* expression pattern in *en>RBF, p35* and in *en>RBF^D253A^* wing discs is clearly different.

**Figure 6:**
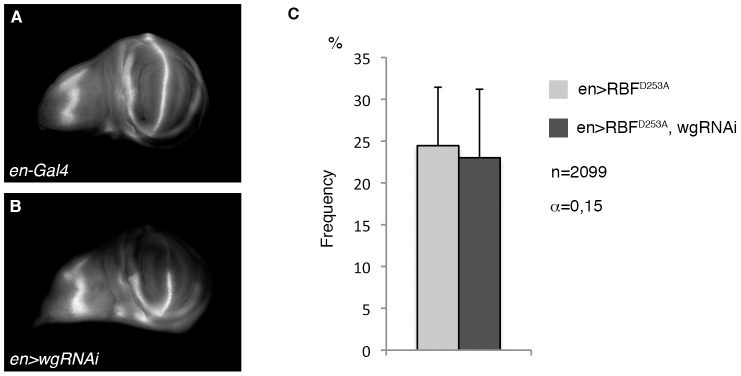
RBF^D253A^-induced overgrowth does not depend on Wg. (A, B) Anti-Wg staining in wing imaginal discs of control (A) or *en-Gal4, UAS-wg-RNAi* third instar larvae (B). No staining is observed in the posterior compartment of discs that express *wg-RNAi*. (C) The frequency of RBF^D253A^-induced ectopic tissue growth is not affected in *UAS-wg-RNAi* co-expressing flies (Chi^2^ test, α = 0.15). All discs are shown with posterior to the top.

We also tested if Wg was required for RBF^D253A^-induced overgrowth by using a *UAS-wg-RNAi* construct. In *en>wg-RNAi* wing discs, the Wg staining observed after immuno-detection was strongly decreased in the posterior compartment, indicating that this *wg-RNAi* construct efficiently abolished *wg* translation (Fig. 6 A, B). We did not observe any significant difference in the frequency of overgrowth phenotype between *vg>RBF^D253A^* and *vg>RBF^D253A^, wg-RNAi* flies (Fig. 6 C). Taken together, these results show that co-expression of *RBF* and *p35* induces hyperplastic proliferation and *wg* ectopic expression as described previously for other pro-apoptotic genes in undead cells. In a distinct manner, RBF^D253A^ expression seems to induce overgrowth that would implicate the JNK pathway but not *wg* ectopic expression. These results suggest that a mutation of the TELD sequence of RBF deregulates apoptosis-induced proliferation, in a JNK-dependent and Wg-independent way.

## Discussion

In this study, we have generated transgenic Drosophila strains expressing a *RBF^D253A^* mutant form under control of the *UAS/*Gal4 system. We observed that this form has an increased pro-apoptotic activity compared to RBF and induces abnormal non-cell autonomous proliferation. This different effect of *RBF^D253A^* cannot be explained by a modulation in level of the full-length RBF protein. In the wing imaginal disc, RBF^D253A^, but not RBF, induces apoptosis in non-proliferative cells of the ZNC. One can hypothesize that RBF^D253A^ is more stable than RBF, and that an increased level of full-length protein in these cells is pro-apoptotic. In this model, excessive wild type RBF would be cleaved in cells of the ZNC in order to maintain a physiological level of this form, preventing an apoptotic effect of accumulated full-length protein. However, we have controlled by western blot that levels of RBF higher than RBF^D253A^ levels do not induce apoptosis in cells of the ZNC (data not shown) which rules out this hypothesis. Moreover, RBF^D253A^ ubiquitous expression is lethal whereas it is not the case even for higher levels of wild-type RBF. Therefore, these results suggest that the mutant form displays specific properties.

Mutation of the TELD site could modify the interactions between RBF and some of its partners and thus modulate its activity. This site is located in the N-terminal domain of the protein. The crystal structure of the Rb N-terminal domain (RbN) has revealed a globular entity formed by two rigidly connected cyclin-like folds [48]. By analogy, we can assume that the TELD sequence of RBF is located in a proteolytically labile linker important for its conformation and its binding to partners.

Furthermore, the effect of RBF^D253A^ on proliferative tissue differs from that of RBF, as it can induce abnormal non-cell autonomous proliferation. We observed that *vg>RBF^D253A^* adult wings present an overgrowth phenotype that was never observed when RBF was expressed, even at high levels. This overgrowth was correlated to some excessive proliferation induced in wing imaginal discs by RBF^D253A^ expression.

We hypothesize that the lethality associated with RBF^D253A^ ubiquitous expression could be a consequence of this excessive proliferation. Altogether, our results show that the TELD site is important for RBF properties, in the control of both apoptosis and apoptosis-induced proliferation.

Our results suggest that the mechanism involved in the RBF^D253A^-induced overgrowth phenotype depends on the JNK pathway as inhibition of this pathway by expressing *bsk-RNAi* or in a *hep^r75^* heterozygous mutant background abrogated almost completely RBF^D253A^-induced overgrowth. Nevertheless, a simple activation of the JNK pathway in the RBF^D253A^-induced overgrowth phenotype is not sufficient to explain the different effects of RBF and RBF^D253A^ on proliferation, as RBF is also able to induce JNK activation but without any overgrowth phenotype. The JNK pathway is involved in both RBF- and RBF^D253A^-induced apoptosis, thus we cannot exclude that the inhibition of RBF^D253A^-induced overgrowth phenotype is a consequence of the decrease of apoptosis in *bsk-RNAi* expressing and *hep^r75^* contexts. Indeed, RBF^D253A^-induced overgrowth phenotype could result from a misregulation of an apoptosis-induced proliferation process. But this cellular process, by which apoptotic cells promote proliferation of surrounding living cells, also depends on the JNK pathway. Similarly in the literature, “undead cells”, which are kept alive by the caspase inhibitor p35 after induction of apoptosis by a pro-apoptotic gene, induce non-cell autonomous proliferation by a persistent activation of the JNK pathway [43], [47], [49], [50], [51]. Since the JNK pathway is essential for RBF^D253A^-induced overgrowth, it is possible that this cleavage-resistant form of RBF possesses an increased ability to activate the JNK in a manner that could enhance the JNK non-apoptotic functions.

Further investigation will be necessary to clarify the consequences of the JNK pathway activation by RBF and RBF^D253A^, and why this activation could lead to different phenotypes. One could also hypothesize that RBF and RBF^D253A^ do not activate the JNK pathway through the same upstream components, which could lead to different effects.

We showed that contrarily to what is observed in the presence of undead cells, RBF^D253A^-induced overgrowth does not require Wg activity. In undead cells, the JNK pathway activation has been shown to lead to ectopic *wg* expression that is responsible for the observed overgrowth [43], [47], [49], [52]. Moreover, the secretion of Wg is not limited to undead cells, but also occurs in “genuine” apoptotic cells [53]. We hypothesized that RBF^D253A^-induced overgrowth could result from a misregulation of apoptosis-induced cell proliferation through Wg signaling. We rejected this hypothesis since the inhibition of *wg* expression with a *wg-RNAi* construct, that in our experiment completely abrogates the detection of wg protein, did not reduce overgrowth. Furthermore, the *wg* expression pattern in *en>RBF^D253A^* wing discs was different form the pattern observed in *en>RBF, p35* discs that contained undead cells and displayed typical ectopic patches of *wg*-expressing cells. In *en>RBF^D253A^* and *ptc>RBF^D253A^* wing discs, more cells seemed to express *wg*, leading to an enlargement or a deformation of the pattern, but we did not observe ectopic patches of *wg* expression. This enlargement could be due to proliferation of *wg*-expressing cells in response to RBF^D253A^ expression, rather than being a cause of this proliferation as it is reported in the presence of undead cells. These results show that RBF^D253A^ is able to induce ectopic proliferation in a JNK-dependent manner that does not require *wg* ectopic expression and that is therefore different from the characterized mechanism induced by the presence of undead cells in a tissue.

Altogether, our data show that RBF^D253A^ expression misregulates tissue homeostasis by inducing hyper-proliferation and overgrowth, in a Wg-independent manner. It has been shown that regulation of tissue homeostasis by compensatory proliferation or proliferation in response to massive damage during development does not require *wg* expression [43], [54]. One could hypothesize that RBF^D253A^-associated overgrowth phenotype could be the reflection of a misregulation of the compensatory proliferation mechanism by strongly enhancing proliferation of neighboring cells. Thus, the identification of RBF^D253A^ partners could provide a new opportunity to discover regulators of compensatory proliferation, which molecular mechanisms have not yet been elucidated. Besides, understanding how RBF^D253A^ can lead to overgrowth could be of great interest to better characterize the tumor suppressor effect of the pocket protein family members.

## Supporting Information

File S1 **Figure S1 in File S1. RBF contains a consensus site of caspase cleavage.** (A) RBF sequence was scanned for potential caspase cleavage site(s) using the CASVM web server (http://www.casbase.org/). This was done with the P14P10′ window (tetrapeptide cleavage sites with ten additional upstream and downstream flanking sequences) which have the highest accuracy. Only one predicted caspase cleavage site was found in RBF: TELD-253. (B) Amino acid sequences alignment of retinoblastoma protein homologs. Amino acid sequences of proteins from *H. sapiens* (top), *C. elegans* (middle), *D. melanogaster* (bottom) were aligned using the Clustal Omega program. Dashes represent gaps in the sequence. Amino acid sequences shown in boxes correspond to consensus caspase cleavage sites. (C) RBF and RBF cleaved forms analysed by Western Blot. Proteins extracts are made from S2 cells transfected with pActine Gal4 vector or pUAS RBF^p76^-HA (RBF^p76^-HA), pUAS RBF-HA (RBF-HA) or pUAS RBF^D253A^-HA (RBF^D253A^-HA) (Effecten kit, Quiagen). 2.10^6^ cells were cryolysed in PBS pH 7.6 and homogenized in buffer containing 50 mM Tris-Cl pH = 7,4, 150 mM NaCl, 1% NP40, 1 mM DTT, AEBSF^SC^. Proteins were separated in 4–12% Bis-Tris polyacrylamide gels according to the manufacturer's instructions (BioRad) and transferred onto PVDF membrane (Millipore). Blots were incubated with mouse anti-HA (HA.11, Covance) and rabbit polyclonal anti-Actin (1∶500, Sigma). Arrow shows wholes RBF forms and dotted-line arrow shows RBF^p76^. **Figure S2 in File S1. Quantification of RBF and RBF^D253A^ protein rates and** ***rbf*** **mRNA.** (A) RBF and RBF^D253A^ protein rates detected by Western blot analysis. Protein extracts were prepared from embryos carrying the *da-Gal4* driver to induce *UAS-RBF* and *UAS-RBF^D253A^* expressions ubiquitously. Three genotypes were tested: *da-Gal4/+* (control), *da-Gal4/UAS-RBF*, *UAS-RBF^D253A^/+; da-Gal4/+* at 25°C. Actin was used as a loading control, and an RBF antibody was used to detect RBF and RBF^D253A^ (rabbit polyclonal anti-RBF,1∶500, Custom antibody, Proteogenix and rabbit polyclonal anti-Actin, 1∶500, Sigma). Immunoreactive bands were detected by Immobilon™ Western Chemiluminescent HRP Substrate (Millipore) with facilities of ChemiDoc MP System (BioRad). (B) Immunoreactive bands were quantified using the Quantity One software. Under these conditions, the level of RBF protein is significantly higher in embryos expressing UAS-RBF and UAS-RBFD253A than in control embryos (asterisk, ANOVA, p = 7.6E-3); furthermore, there is no significant difference between RBF and RBFD253A protein expression levels (ANOVA, p = 0.48). (C) Quantification of *rbf* mRNA by RT-qPCR in wing imaginal discs. Fifty wing imaginal discs per genotype were dissected on ice. Total RNAs were extracted from each sample using the RNeasy Mini kit (QIAGEN), RT was performed on each sample using random primer oligonucleotides (Invitrogen) with Recombinant Taq DNA Polymerase (Invitrogen). Real-time PCR analysis was performed using the C1000 Touch™ Thermal cycler (Biorad). Data are normalized against *rp49* and correspond to the mean of three independent experiments. Error bars are the S.E.M. Asterisks indicate statistical significant difference between two genotypes (Student test, p<0,05). **Figure S3 in File S1. RBF^D253A^ is pro-apoptotic in the ZNC and induces more apoptosis than RBF in third instar larvae wing imaginal discs.** (A, E) *C96-Gal4* and *vg-Gal4* expression patterns are visualized by *UAS-mtGFP* expression in third instar larvae wing imaginal discs. (B–D, F–H) Apoptotic cells are labeled with Acridine Orange in wing imaginal discs (2 min in 100 ng/ml AO, Molecular Probes); specific staining of apoptotic cells corresponds to bright white patches. (B, F) *C96-Gal4*/+ and *vg-Gal4*/+ control discs have few apoptotic cells. (C) *C96-Gal4/UAS-RBF* wing discs are similar to control. (D) Some apoptotic cells are observed within the *C96-Gal4* expression domain in *UAS-RBF^D253A^/X*; *C96-Gal4*/+ discs (white arrows). (G, H) Apoptotic cells are observed within the *vg-Gal4* expression domain in *vg-Gal4*/+; *UAS-RBF*/+ and *UAS-RBF^D253A^/X; vg-Gal4*/+ wing discs (white arrows). (G) The white arrowhead indicates a zone at the center of the pouch where cells are not AO-labeled in *vg-Gal4/+; UAS-RBF*/+ wing discs. All discs are shown with posterior to the top. Discs were mounted in AO and observed with a conventional Leica DMRHC research microscope using the L5 filter to detect AO fluorescence. **Figure S4 in File S1. *RBF^D253A^* expression at the antero-posterior boundary of wing imaginal discs induces more apoptosis than RBF, but adult phenotypes are similar.** (A–C) Distances between veins 3 and 4 (dv3-v4) were measured at the posterior third of the wings using the Adobe Photoshop CS3 software, as indicated by the black lines. 20 wings were measured to estimate the average distance between veins 3 and 4 (dv3-v4±s.e.m) for each genotype: (A) *ptc-Gal4*/+ control wings, (B) *ptc-Gal4/UAS-RBF* flies, (C) *UAS-RBF^D253A^; ptc-Gal4* flies. *UAS-RBF* as well as *UAS-RBF^D253A^* expression under the control of *ptc-Gal4* brings veins 3 and 4 closer. (D) *ptc-Gal4* expression pattern is visualized by *UAS-mtGFP* expression in third instar larvae wing imaginal discs. Apoptotic cells are labeled with TUNEL (E–G) or Acridine Orange (H–J) in the wing imaginal discs; specific staining of apoptotic cells corresponds to bright white patches. (E, H) Few apoptotic cells are observed in *ptc-Gal4*/+ control discs. (F, G, I, J) In *ptc-Gal4/UAS-RBF* and *UAS-RBF^D253A^*/+; *ptc-Gal4*/+ discs, apoptotic cells are observed within the *ptc-Gal4* expression domain (white arrows). More apoptotic cells are observed in *UAS-RBF^D253A^*/+; *ptc-Gal4*/+ discs. All discs are shown posterior to the top. Discs were observed with a conventional Leica DMRHC research microscope using the L5 filter to detect AO fluorescence and using the N2.1 filter to detect TUNEL. **Figure S5 in File S1. RBF^D253A^ expression at the antero-posterior boundary of wing discs alters the Wg pattern.** (A–C) anti-Wg (green) and anti-RBF (red) staining with enlarged views of boxed areas. (A) Wg pattern in control *ptc-Gal4*/+ wing disc. (B) *ptc-Gal4/UAS-RBF* discs have the same Wg pattern than control discs. (C) In *UAS-RBF^D253A^/X; ptc-Gal4*/+ discs, the Wg pattern is altered (white arrowhead) when compared to control discs. More *wg* expressing cells adjacent to the RBF^D253A^ expression domain in the posterior compartment are observed. All discs are shown with posterior to the top. Discs were observed with a conventional Leica DMRHC research microscope using the L5 filter to detect Wg-associated fluorescence and using the N2.1 filter to detect RBF-associated fluorescence. **Figure S6 in File S1. Expression of *RBF^D253A^* and co-expression of *RBF* and *p35* lead to different Wg patterns.** (A–C) RBF immuno-staining (red). (D–F) anti-Wg immuno-staining (green). (D) Control Wg pattern in *UAS-p35/X; en-Gal4*/+ wing disc. (E) In *UAS-p35/X; en-Gal4/+; UAS-RBF*/+ wing discs, the Wg pattern is altered when compared to control discs: ectopic patches are observed in the posterior compartment (arrows and box b). (F) In *UAS-RBF^D253A^/X; en-Gal4*/+ wing discs, the Wg pattern is altered when compared to the control, and an enlargement of this pattern is observed in the posterior compartment (box a). All discs are shown with posterior to the top. Discs were observed with a conventional Leica DMRHC research microscope using the L5 filter to detect Wg and the N2.1 filter to detect RBF associated fluorescence.
(ZIP)
