# Supplementary material for: Mutating RBF Can Enhance Its Pro-Apoptotic Activity and Uncovers a New Role in Tissue Homeostasis
Source: PLoS One. 2014 Aug 4;9(8):e102902. doi: 10.1371/journal.pone.0102902 (PMC4121136; doi:10.1371/journal.pone.0102902)

A

CASVM: Server for SVM Prediction of Caspase Substrates Cleavage Sites

Server Results

Your input sequence has been scanned for caspase cleavage sites. Results are reported below:

**Sequence Name**	RBF
**Sequence**	MSEPD...GEHET
**Length of Sequence**	845 residues
**Potential Sites**	SEPD-5<br>ATSD-22<br>TSDD-23<br>LCRD-38<br>LNMD-42<br>QWCD-123<br>EIED-137<br>EDLD-139<br>IIMD-157<br>IKLD-190<br>KLDD-191<br>NTVD-210<br>CCID-223<br>KRTD-236<br>TELD-253<br>NFCD-267<br>GNKD-298<br>GEFD-331<br>SAYD-339<br>ALND-348<br>RKQD-363<br>MAGD-373<br>EPTD-399<br>YLLD-448<br>NKPD-460<br>PDID-462<br>WVLD-503<br>VNLD-576<br>LMKD-631<br>RHLD-635<br>RMED-653<br>KFSD-658<br>VFID-680<br>INED-684<br>KVKD-691<br>FVID-710<br>VTPD-717<br>RASD-724<br>PAKD-775<br>SFGD-794<br>AVMD-817<br>VMDD-818
**Predicted Sites** (P4-P1)	Not selected
**Predicted Sites** (P4-P2')	Not selected
**Predicted Sites** (P14-P10')	TELD-253

B

```
RYQEYIRRIQEGLAVSCVIFKKFCRIFCKIFEEIKVGS-------------ENCPSSHEL  280
KVDNAMSRLLKKYDVLFALFSKLERTCELIYLTQPSSS------------ISTEINSALV  190
EFVNEIEDLDRKFSITFMLHKRFRIIMDMIFSCPPNEKKHSKYISLHGNHAHGKCSYIKL  189

FTVLWTSFLVMKSRM--TVDDLISNYQLLFSILDQVYTEMCSMK--EGIVHHLN------  330
LKVSWITFLLAKGEVLQMEDDLVISFQLMLCVLDYFIKLSPPMLLKEPYKTAVI-PINGS  249
DDICWRLFLCAKNQKPSNTVDLVTSYNLMICCIDLIYNNVLA----EKRTDLINPKFEGL  245

----------QKFVEDLLENDCTIIRALCTQFGGSVLD-ARHF---SDHTFKKM------  370
PRTPRRGQNRSARIAKQLENDTRIIEVLCKEHECN-------IDEVKNVYFKNFIPFM--  300
PSNWTEL---------DFRHNPHCILSNFCDMTEEAKAMKATTFRQIMSSFFQASTIYGNK  297

-EKTGIPSTWNFQEFRDLIMNVPKTAYENYLLQRGSIDERIFIPSVEDFSKIFQSPDTYS  429
-NSLGLVTSNGL-----PEVENLSKRYEEIYLKNKDLDARLFLDHDKTLQTDS--IDSFE  352
DTMLGLLANENF---E-RNLKSLNISYEQYVLSVGEFDERILSAYDAGEHTAL-------  346
```

C

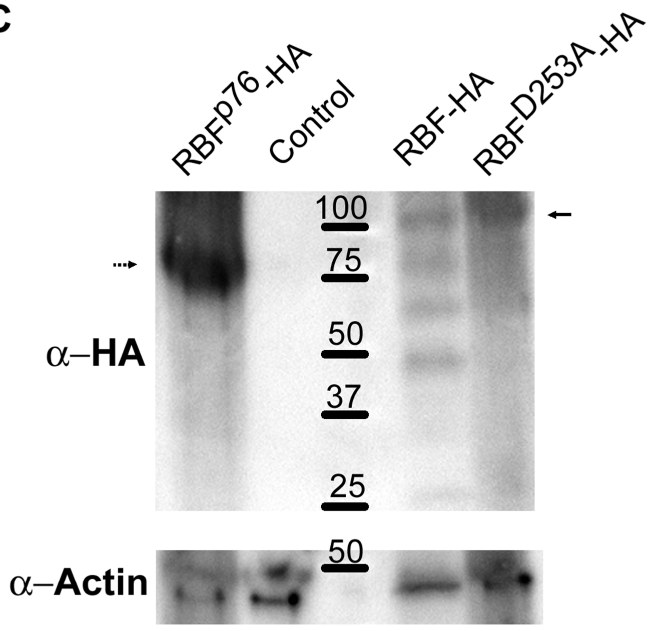

Supplement: File S1 — Figure S1 in File S1. RBF contains a consensus site of caspase cleavage. (A) RBF sequence was scanned for potential caspase cleavage site(s) using the CASVM web server (http://www.casbase.org/). This was done with the P14P10′ window (tetrapeptide cleavage sites with ten additional upstream and downstream flanking sequences) which have the highest accuracy. Only one predicted caspase cleavage site was found in RBF: TELD-253. (B) Amino acid sequences alignment of retinoblastoma protein homologs. Amino acid sequences of proteins from H. sapiens (top), C. elegans (middle), D. melanogaster (bottom) were aligned using the Clustal Omega program. Dashes represent gaps in the sequence. Amino acid sequences shown in boxes correspond to consensus caspase cleavage sites. (C) RBF and RBF cleaved forms analysed by Western Blot. Proteins extracts are made from S2 cells transfected with pActine Gal4 vector or pUAS RBFp76-HA (RBFp76-HA), pUAS RBF-HA (RBF-HA) or pUAS RBFD253A-HA (RBFD253A-HA) (Effecten kit, Quiagen). 2.106 cells were cryolysed in PBS pH 7.6 and homogenized in buffer containing 50 mM Tris-Cl pH = 7,4, 150 mM NaCl, 1% NP40, 1 mM DTT, AEBSFSC. Proteins were separated in 4–12% Bis-Tris polyacrylamide gels according to the manufacturer's instructions (BioRad) and transferred onto PVDF membrane (Millipore). Blots were incubated with mouse anti-HA (HA.11, Covance) and rabbit polyclonal anti-Actin (1∶500, Sigma). Arrow shows wholes RBF forms and dotted-line arrow shows RBFp76. Figure S2 in File S1. Quantification of RBF and RBFD253A protein rates and rbf mRNA. (A) RBF and RBFD253A protein rates detected by Western blot analysis. Protein extracts were prepared from embryos carrying the da-Gal4 driver to induce UAS-RBF and UAS-RBFD253A expressions ubiquitously. Three genotypes were tested: da-Gal4/+ (control), da-Gal4/UAS-RBF, UAS-RBFD253A/+; da-Gal4/+ at 25°C. Actin was used as a loading control, and an RBF antibody was used to detect RBF and RBFD253A (rabbit polyclonal anti- [file pone.0102902.s001.zip › Figure S1.pdf]
